# Phlegmasia Cerulea Dolens Secondary to COVID-19 and May-Thurner Syndrome: A Case Report

**DOI:** 10.7759/cureus.21301

**Published:** 2022-01-16

**Authors:** Lamia Alghamdi, Nashwan Alattab, Abdullah Alwohaibi, Yazeed H Alotaibi, Mohammed AlSheef

**Affiliations:** 1 Internal Medicine, King Fahad Medical City, Riyadh, SAU; 2 Vascular Surgery, King Fahad Medical City, Riyadh, SAU

**Keywords:** catheter-directed thrombolysis, deep vein thrombosis (dvt), covid 19, may-thurner's syndrome, phlegmasia cerulea dolens

## Abstract

Coronavirus disease 2019 (COVID-19) is associated with significant thromboembolic risk. Extensive deep vein thrombosis can infrequently progress to phlegmasia cerulea dolens that carries high morbidity and mortality rates. We report a case of a middle-aged male presenting with phlegmasia cerulea dolens in the context of COVID-19 and underlying May-Thurner syndrome, associated with transiently positive antiphospholipid antibodies.

## Introduction

Severe acute respiratory syndrome coronavirus 2 (SARS-CoV-2) was first identified in Wuhan, China, in early December 2019. The clinical presentation of coronavirus disease 2019 (COVID-19) is variable, ranging from a mild, self-limited, flu-like illness to life-threatening pneumonia, acute respiratory distress syndrome (ARDS), systemic inflammation, and multiorgan failure [1-2]. COVID-19 is associated with both arterial and, more particularly, venous thromboembolic events [3]. The risk of thrombotic complication is associated with high D-dimer, C reactive protein (CRP), erythrocyte sedimentation rate, and ferritin levels at presentation [3].

May-Thurner Syndrome (MTS) is characterized by the compression of the left common iliac vein by the right common iliac artery, leading to a predisposition to extensive deep venous thrombosis (DVT) of the left lower limb [4]. MTS is typically managed surgically by endovascular methods to correct the anatomical anomaly and associated thrombosis [4-5].

Phlegmasia cerulea dolens (PCD) is a rare but serious complication of DVT, characterized by severe pain, swelling, and cyanosis of the affected limb [6]. Early recognition and prompt intervention are critical, as delay in treatment may result in arterial ischemia and, eventually, limb gangrene. PCD is associated with high amputation rates, pulmonary embolism, and mortality [7].

We are reporting a case of DVT complicated by PCD in a male with COVID-19 and underlying MTS, associated with transiently positive antiphospholipid antibodies.

## Case presentation

A 58-year-old male with no prior medical conditions presented to our emergency department complaining of severe pain and blue discoloration of the left lower limb. The onset of symptoms was three days prior to presentation. The delay in the presentation was due to the reluctance of the patient to seek medical care during the nationwide lockdown amid the pandemic in Saudi Arabia. The patient reported having dyspnea and cough for three days. He reported no fever, chills, chest pain, hemoptysis, syncope, orthopnea, paroxysmal nocturnal dyspnea, diarrhea, nausea, or vomiting. A review of other systems was negative. There was no history of smoking, recent travel, surgery or immobilization, current or prior malignancy, and no family history of VTE or connective tissue disease. The patient presentation was prior to the approval of COVID-19 vaccines.

**Figure 1:**
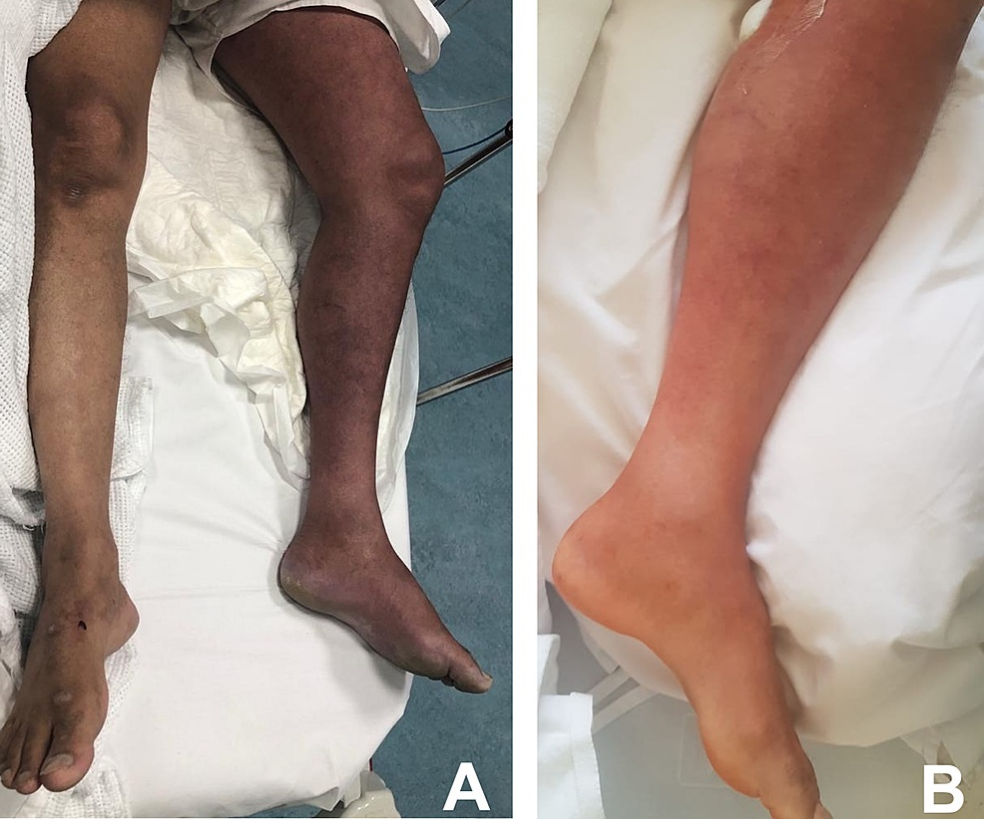
The appearance of the left leg at presentation (A) and 10 hours after catheter-directed thrombolysis (B)

On examination, the patient was hemodynamically stable with a blood pressure of 127/79 mmHg, heart rate of 71 bpm, a temperature of 36.6 C, and oxygen saturation of 97% breathing ambient air. Examination of the left lower limb showed a remarkable swelling and bluish discoloration extending up to the thigh compared to the right lower limb (Figure 1A). On palpation, the left leg was tender and cool. The femoral pulse was palpable; however, the popliteal, posterior tibial, and dorsalis pedis pulses were not palpable. Bedside Doppler examination confirmed the absence of pulses of the popliteal, posterior tibial, and dorsalis pedis arteries. Chest examination was significant for bilateral rales. The remainder of the examination, including the cardiovascular and abdominal examinations, were unremarkable.

**Figure 2:**
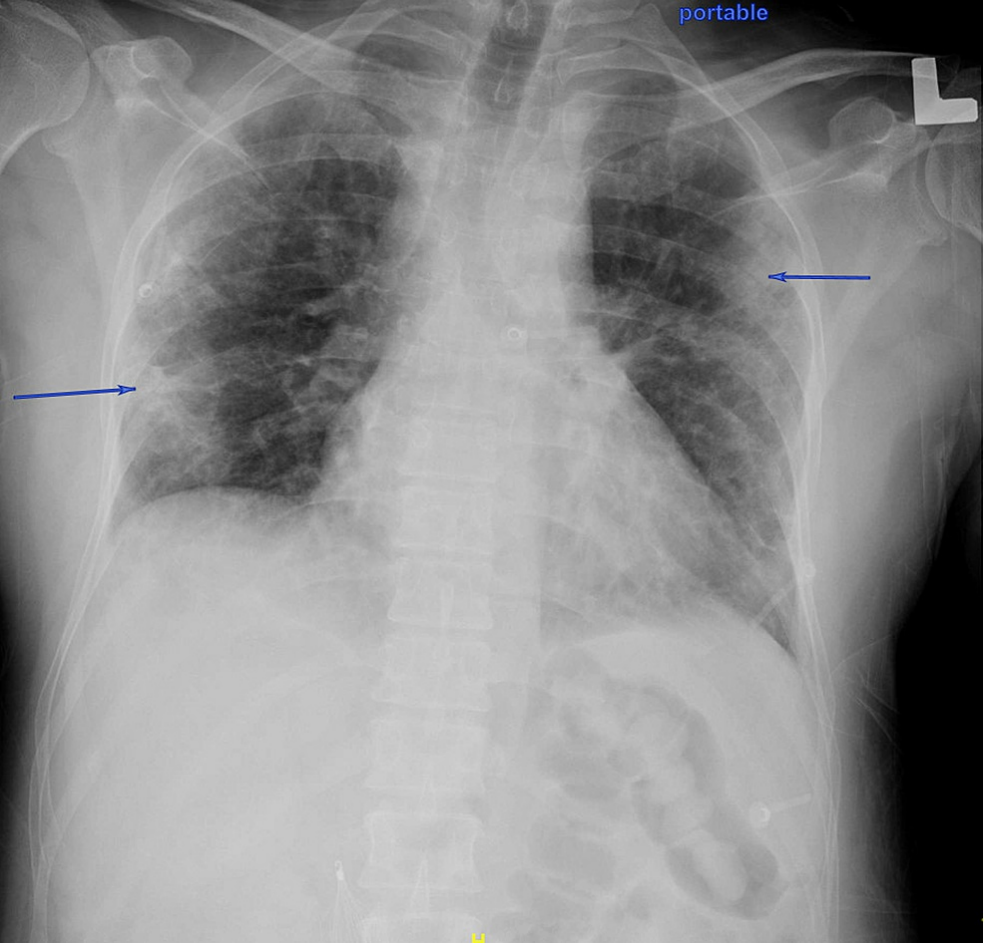
Chest X-ray at presentation Blue arrows: Peripheral bilateral patchy opacities

Initial laboratory test results showed leukocytosis with a neutrophilic predominance and a normal platelet count, low fibrinogen, and elevated INR and D-dimer (Table 1). Nasopharyngeal swab for SARS-CoV-2 was positive. Chest X-ray showed bilateral peripheral patchy opacities (Figure 2).

**Table 1:** Laboratory results on initial presentation ALT: alanine transaminase; AST: aspartate aminotransferase

Patient results		Reference range
White cell count (10e9/L)	19.9	3.9 - 11
Neutrophils (%)	86.5	30 - 70
Lymphocytes (%)	8.00	23 - 60
Absolute neutrophil count (10e9/L)	17.25	1.35 - 7.5
Absolute lymphocyte count (10e9/L)	1.59	1.5 - 4.3
Hemoglobin (g/dL)	15.00	11 - 16
Platelets count (10e9/L)	247	155 - 435
Prothrombin time (S)	17.400	9.7 - 12.6
Activated partial thromboplastin time (S)	28.300	25.3 - 38.3
International normalized ratio (INR)	1.46	0.81 - 1.23
Fibrinogen (g/L)	0.39	1.61 - 4.39
Troponin (ng/L)	6.3	0 - 15.6
Lactate (mmol/L)	3.5	0.5 - 2.2
D-dimer (µg/mL)	128	0 - 0.5
Lactate dehydrogenase (U/L)	904	125 - 220
Ferritin (ng/mL)	883	10 - 204
C-reactive protein (mg/L)	109	1 - 3
ALT (U/L)	60	0 - 55
AST (U/L)	92	5 - 34
Alkaline phosphatase (U/L)	79	40 - 150
Total bilirubin (umol/L)	14	3 - 20
Creatine kinase (U/L)	3473	30 - 200
Creatinine umol/L	68	64 - 104
Potassium (mmol/L)	5.1	3.5 - 4.5
Corrected calcium (mmol/L)	2.21	2.1 - 2.55
Phosphate (umol/L)	0.96	0.74 - 1.52
Magnesium (mmol/L)	0.80	0.66 - 1.07

Ultrasound of the left lower limb showed an echogenic thrombus with a lack of blood flow and compressibility in the left external iliac, common femoral, and superficial femoral veins. Partial thrombosis of the popliteal vein was also noted.

**Figure 3:**
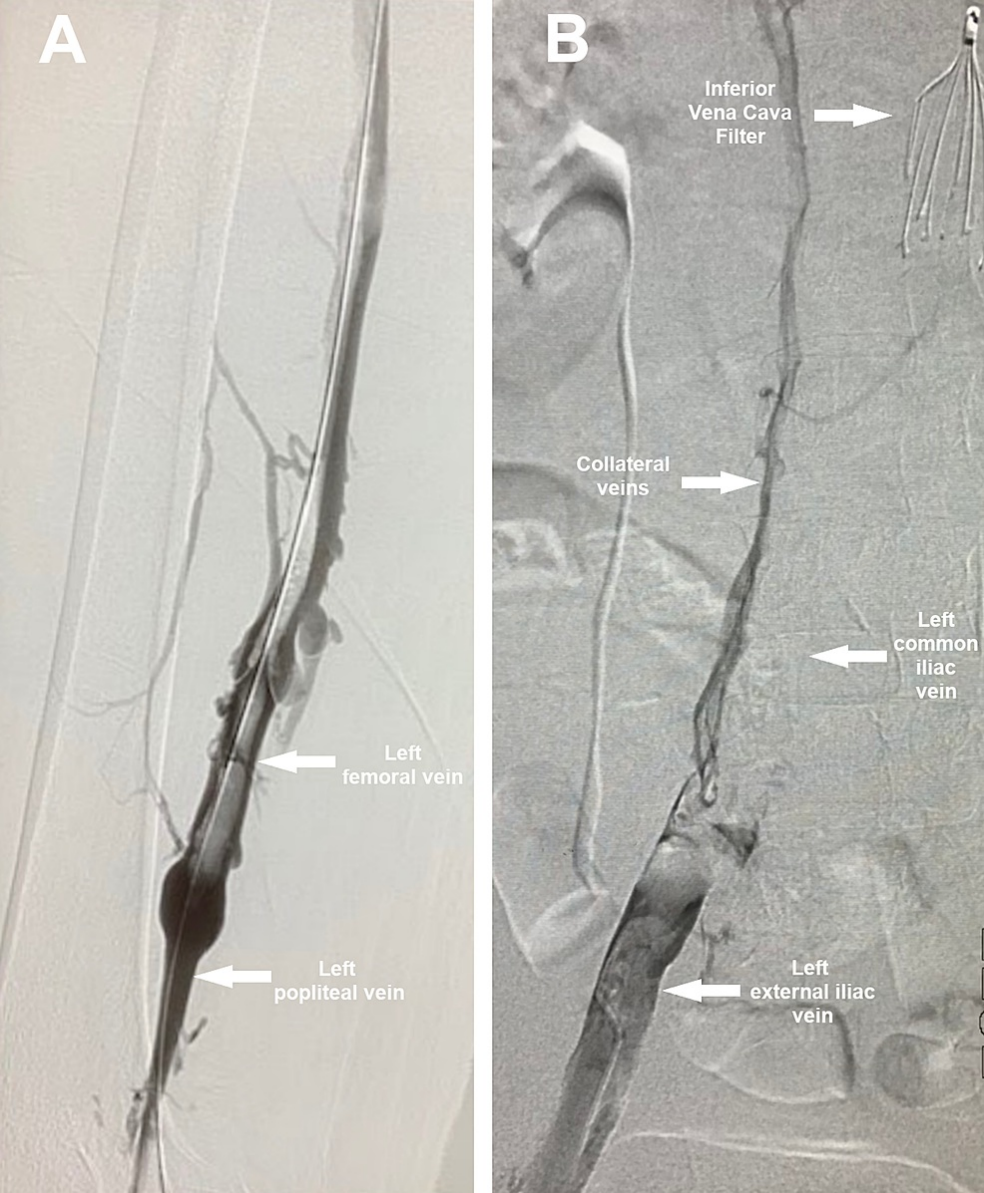
Venogram performed after IVC filter insertion showing extensive thrombosis involving the femoral (A), left external iliac, and left common iliac veins, with the formation of collaterals (B) IVC: inferior vena cava

Based on these findings, we diagnosed the patient with extensive DVT complicated by PCD. We started heparin infusion and consulted the vascular surgery team for urgent intervention. First, an inferior vena cava (IVC) filter was inserted below the renal vein under ultrasound and fluoroscopy guidance. Then, the left popliteal vein was accessed; a diagnostic venogram (Figure 3) revealed thrombosis of the common femoral, external, and common iliac veins reaching up to the IVC, where a floating thrombus was found. A catheter was inserted reaching the proximal site of the thrombus and 10 ml of alteplase was injected. The catheter was fixed to provide continuous thrombolytic therapy for 12 hours in the intensive care unit. Following catheter-directed thrombolysis, an examination revealed marked improvement in swelling and discoloration (Figure 1B); the peripheral pulses were present on palpation and audible on bedside Doppler.

**Figure 4:**
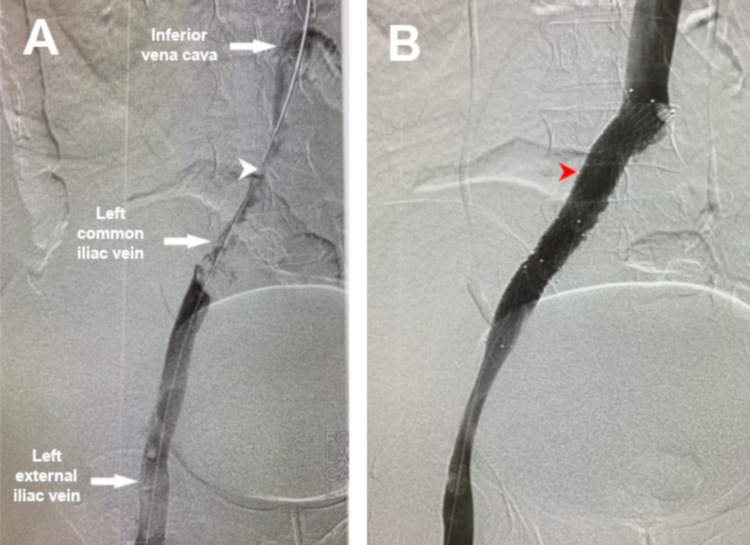
Venogram performed the next day of presentation after catheter-directed thrombolysis showing: A) thrombosis involving left common iliac vein and stenosis and the junction with the right common iliac artery; B) Venogram after mechanical thrombectomy and stenting of the left common iliac vein White arrowhead: Point of compression of the left common iliac vein by the right common iliac artery. Red arrowhead: stent placed in the left common iliac vein

The following day, venography was performed, which showed thrombosis in the femoral and iliac veins and the IVC confluence. Aspiration was done using AngioJet (Boston Scientific, Marlborough, MA); the subsequent venogram showed resolution of thrombus, elucidating narrowing of the left common iliac vein consistent with May-Thurner Syndrome (Figure 4A). Following that, the iliac vein was stented and the final venogram showed patent veins with adequate blood flow (Figure 4B). Twelve days later, the IVC filter was removed. During hospitalization, the patient was on a therapeutic dose of low-molecular-weight heparin (enoxaparin 80 mg subcutaneously twice daily). The thrombophilia workup was negative (Table 2) except for elevated beta-2 glycoprotein IgM levels.

**Table 2:** Thrombophilia workup Ig: immunoglobulin

	Patients result	Reference range
JAK2 V617F mutation	Normal	
Factor II (prothrombin)	Normal	
Factor V Leiden	Normal	
Antithrombin III (%)	95	84-117
HLA B51	Negative	
Lupus anticoagulant	Negative	
B2 glycoprotein IgG (U/mL)	2.1	< 20
B2 glycoprotein IgM (U/mL)	328	< 20
Anti-cardiolipin IgG (U/mL)	2.1	< 20
Anti-cardiolipin IgM (U/mL)	12.2	< 20

After 14 days of hospitalization, the patient was discharged on enoxaparin 80 mg twice daily to complete three months of anticoagulation. The patient was followed up in the clinic twice, three and five months later, he was mobilizing independently, and his affected limb was entirely back to normal with no significant swelling, discoloration, or pain. Repeated beta-2 glycoprotein IgM showed normal levels of 7.8 U/mL.

## Discussion

PCD is a rare but serious complication of DVT. It occurs due to extensive thrombosis involving deep veins and their collaterals, resulting in venous congestion and arterial insufficiency, predisposing patients to rhabdomyolysis, limb ischemia, and gangrene. Furthermore, venous hypertension leads to increased intra-compartmental pressure of the leg, which leads to lymphatic obstruction initially, which aggravates further increased intra-compartmental pressure; this will eventually cause arterial occlusion at the capillary level. Reported mortality rates range from 25% to 40% [8]. Initial medical management includes anticoagulation, limb elevation, analgesics, and intravenous fluids. There is no consensus on the management of PCD; therapeutic modalities reported in the literature include catheter-directed thrombolysis, percutaneous thrombectomy, and surgical thrombectomy [7-10]. The goal of therapy is to reduce the thrombus burden and prevent thrombus propagation. The IVC filter is often used to reduce the risk of embolization. In this case, the patient received catheter-directed thrombolysis and an IVC filter followed by percutaneous mechanical thrombectomy using AngioJet and stent insertion. The patient was treated with enoxaparin 80 mg twice daily to complete three months of anticoagulation. Direct oral anticoagulants (DOAC) (rivaroxaban, apixaban, and edoxaban) are certainly of more practical use than vitamin K antagonists (warfarin), especially during COVID-19 pandemic, as they do not need laboratory monitoring. However, due to positive antiphospholipid antibodies (APLA), we elected to use enoxaparin as direct oral anticoagulants (DOACs) are not recommended in patients with positive APLA due to high recurrence rates of thromboembolic events [11]. Warfarin was not used as frequent INR monitoring potentially exposes patients to COVID-19 during the peak of the COVID-19 pandemic.

MTS is a syndrome of venous outflow obstruction caused by extrinsic compression of the left common iliac vein by the right common iliac artery with subsequent partial obstruction of the left common iliac vein. Patients with MTS are predisposed to extensive left iliofemoral DVT. Although this syndrome is rare, its incidence and prevalence are probably underestimated due to the lack of awareness about the disease [12]. Many patients with MTS are asymptomatic or mildly symptomatic until provoked by another condition predisposing them to a hypercoagulable state such as covid-19 as in our patient.

Most reported cases of PCD are in patients with underlying malignancy or thrombophilia [10]. In this case, the hypercoagulable state was likely secondary to COVID-19 infection with underlying MTS. Since the start of the pandemic, several case reports of PCD in COVID-19 have been published in patients without a history of venous thromboembolism or predisposing conditions [13-15] and in patients with pre-existing thromboembolic conditions [16-17]. However, this is the first reported case of a patient with underlying MTS.

In this case, whether the transiently elevated antiphospholipid antibodies (APLA) play any role in the pathogenesis of the extensive thrombosis is uncertain. Viral illnesses have been known to be associated with transiently elevated APLA [18]; infections with SARS-CoV-2 were shown to be associated with transiently elevated APLA [19-20]; the clinical significance of these findings remains unknown.

## Conclusions

COVID-19 is recognized as a major thromboembolic risk factor. As the disease continues to infect millions worldwide, it is crucial for health care providers to recognize PCD early and intervene timely to reduce morbidity and mortality. It is also essential to address the classical risk factors of thromboembolism such as inherited and acquired thrombophilia, malignancies, and MTS. More research is needed to aid in determining the standard of care of PCD.
